# Estimation and sensitivity analysis of fouling resistance in phosphoric acid/steam heat exchanger using artificial neural networks and regression methods

**DOI:** 10.1038/s41598-023-44516-6

**Published:** 2023-10-19

**Authors:** Rania Jradi, Christophe Marvillet, Mohamed Razak Jeday

**Affiliations:** 1 Research Laboratory «Process, Energy, Environment and Electrical Systems», National Engineering School of Gabès, Gabès, Tunisia; 2 CMGPCE Laboratory, French Institute of Refrigeration (IFFI), National Conservatory of Arts and Crafts (CNAM), Paris, France

**Keywords:** Chemical engineering, Mathematics and computing

## Abstract

One of the most frequent problem in phosphoric acid concentration plant is the heat exchanger build-up. This problem causes a reduction of the performance of this equipment and an increase of energy losses which lead to damage the apparatus. In this study, estimation of fouling resistance in a cross-flow heat exchanger was solved using a linear [Partial Least Squares (PLS)] and non linear [Artificial Neural Network (ANN)] methods. Principal Component Analysis (PCA) and Step Wise Regression (SWR) were preceded the modeling in order to determine the highest relation between operating parameters with the fouling resistance. The values of correlation coefficient (r^2^) and predictive ability which are equal to 0.992 and 87%, respectively showed a good prediction of the developed PLS model. In order to improve the results obtained by PLS method, an ANN model was developed. 361 experimental data points was used to design and train the network. A network containing 6 hidden neurons trained with Broyden–Fletcher–Goldfarb–Shanno (BFGS) algorithm and hyperbolic tangent sigmoid transfer function for the hidden and output layers was selected to be the optimal configuration. The Garson’s equation was applied to determine the sensitivity of input parameters on fouling resistance based on ANN results. Results indicated that acid inlet and outlet temperatures were the high relative important parameters on fouling resistance with importance equal to 56% and 15.4%, respectively.

## Introduction

The supply of heat is a vital step in production chains for almost all industrial activities. This supply is generally carried out by various equipment such as heat exchangers^1^.

The functioning of these equipment is made by two modes of heat transfer as either directly, where two fluids exchange heat between them without any separation, or indirectly where the hot fluid gives up its heat through a material that separates it from the cold fluid^2^.

In the aim to better suit their various applications, heat exchangers are widely used in industry in different configurations and sizes. Several mechanism can affect the proper functioning of these equipment. The major mechanism is the phenomenon of dirt deposition on the heat exchange walls of heat exchangers. This phenomenon is commonly known as fouling^3^. It is defined as the accumulation of any unwanted deposit such as crystalline, biological, particulate or chemical reaction product on the surface of the heat exchanger. This phenomenon has an adverse impact on the thermal and hydraulic performances of the heat exchanger^4,5^. The presence of this deposit on heat exchanger surface causes an additional thermal resistance which leads to reducing heat transfer efficiency^6^. The fouling layer can cause also erosion of heat exchanger surfaces and may even cause a catastrophic failure of heat exchanger^2^. Fouling deposition tends to reduce the free space for flow movement, which degrades the hydraulic performance and can include additional problems such as higher maintenance costs for removal of fouling deposits and replacement of corroded equipment^2,7^.

To this day, fouling remaining the main unresolved problem in heat transfer and an almost universal problem in the design and operation of heat exchanger equipment. Several factors can influence the formation of fouling in the heat exchanger such as the operating parameters, fouling fluid properties and design parameters of the heat exchanger^6^.

Recently, the application of proficient methods are used to counter this problem. Among these methods, artificial neural networks are used in order to establish a relation between affecting factors of the process as input variables and fouling resistance as output variable^1,8,9^. Artificial neural networks is a technique that can provide useful tools for modeling and correlating practical heat transfer problems. One of the most advantages of this method is their ability to learn massive amounts of data^10^.

By using ANN approach, Jradi et al. predicted fouling resistance in cross flow^6,9^ and in shell and tube^1^ heat exchanger in order to plan suitable cleaning schedules. Besides, the ANN method was used by Jradi. et al. to develop the predictive models to estimate the fouling resistance in order to predict a cleaning schedule and to control operation of the phosphoric acid concentration plant^7^.

Another mentionable approach to depict the relation between inputs and output variables is Partial Least Square (PLS) regression. PLS is a powerful statistical parameter tool which can explore the mathematical correlation between input and output variables based on input matrix^7^. It is used in situations where a response is influenced by several independent variables.

The combining approaches (PLS and ANN) were successfully applied by several researchers in the field of processes modeling. The performance of these two methods was evaluated and compared for hardness modeling during the ripening process of Swiss-type cheese using spectral profiles^11^ and for the quantitative analysis of quartz in the presence of mineral interferences^12^.

In the field of fouling modeling, from the experimental data, Jradi et al. developed accurate and reliable models of fouling phenomenon using PLS and ANN methods, in order to make a comparative study based on some statistical indices among these different models to the modeling, and the losses prediction of heat exchangers performances due to the fouling phenomenon^7^.

Recently, Principal Component Analysis (PCA) which precede PLS method is used to determine the effect of operating parameters on the output parameter^13,14^.

In this research, PLS and ANN methods were developed using data set collected from phosphoric acid concentration plant. Before undertaking modeling, PCA and SWR were used to study the variability of the system through the identification of relations between the collected variables in addition to outliers detection. Two accurate measurements (mean squared error (MSE) and correlation coefficient (r^2^)) were used to evaluate and to compare the developed models with experimental data. Moreover, the relative importance of each input parameters on fouling resistance was determined using Garson's equation and based on ANN results.

## Material and methods

### Experimental process

**Figure 1:**
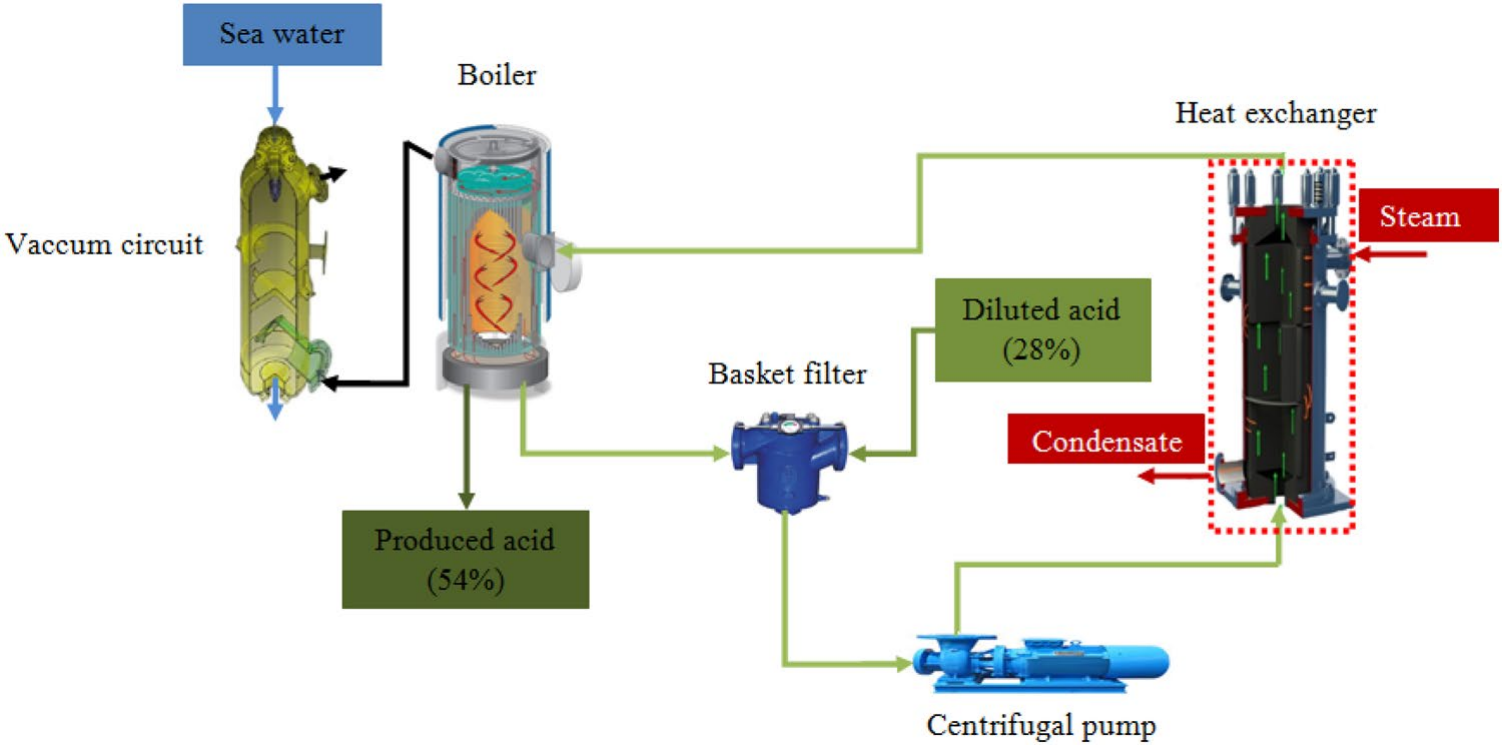
Schematic drawing of phosphoric acid concentration plant.

Figure 1 presents a schematic drawing of the phosphoric acid concentration plant in Chemical Tunsian Group in Gabes (Tunisia). It consists of five equipment which are: a basket filter, a centrifugal pump, a cross flow heat exchanger, a boiler and a condenser^15^.

- *Basket filter* The dilute phosphoric acid (28% P_2_O_5_) and the circulating phosphoric acid (the undesired output which came from a piping system inside the boiler) blendes at the basket filter. This equipment is used to retain crusts and gypsum debris contained in the blending formed. Otherwise, if these impurities are not retained by the basket filter, they may damage the circulation pump as well as the heat exchanger tubes.
- *Centrifugal pump* The acid, free of coarse impurities, is send through the centrifugal pump to the heat exchanger.
- *Cross flow heat exchanger* The heat exchanger allows to raise the acid temperature from about 70 °C to about 80 °C via steam.
- *Boiler* The superheated mixture of acid exiting the heat exchanger undergoes evaporation at the boiling point in the boiler with the aim of reaching the desired product concentration (54% P_2_O_5_).
- *Condenser* The main function of this equipment is to reduce the incurring non-condensable gases coming out from the boiler and also to reduce the amount of heat supplied by the heat exchanger.

### Data collection and calculation procedure

Several operating data were collected from the phosphoric acid concentration unit for a period of 1 year. A total of 361 observations containing 7 operating cycles and 6 variables were gathered^16^. The parameters collected are classified into two major groups as shown in Table 1:

- Thermal operating parameters
- Hydraulic operating parameters

**Table 1:** Ranges of collected data.

Group	Variable	Unit	Min	Max	Uncertainties
Thermal	Acid inlet temperature	°C	68	78	± 0.3 °C
	Acid outlet temperature	°C	77	86.8	± 0.3 °C
	Steam temperature	°C	116	125	± 0.3 °C
Hydraulic	Acid density	Kg/m^3^	1620	1656	± 0.05%
	Suction pressure	bar	0.85	1.25	± 1.6%
	Discharge pressure	bar	3.1	3.9	± 1.6%
	Time	h	0	122	

The first group includes inlet and outlet temperatures of cold fluid and temperature of hot fluid which were measured from the two extremities of the heat exchanger.

The second group includes suction and discharge pressure which were measured in the two extremities of the centrifugal pump. Moreover, it contains acid density which was measured in the inlet of the heat exchanger of the cold fluid.

Each parameter of the two group was measured every 2 h in the phosphoric acid concentration unit. In addition, the parameter time is also essential in the prediction of the fouling resistance. It is used for the cleaning schedule prediction. The ranges of these data is given in Table 1^9^.

The data set collected were used to calculate the fouling resistance. The calculation procedure was carried out by using the following equations^7^:

1 $$Rf = \frac{1}{{U_{fouling} }} - \frac{1}{{U_{clean} }}$$

where Rf and U are the fouling resistance and overall heat transfer coefficient, respectively.

$${\text{U}}_{{{\text{fouling}}}} = {\text{ U}}\left( {\text{t}} \right){\text{ and U}}_{{{\text{clean}}}} = {\text{ U}}\left( {{\text{t}} = 0} \right)$$

2 $$U_{fouling} = \frac{{\dot{v}_{ac,cir} \times \rho_{ac} \times Cp_{ac} \times \left( {T_{out,ac} - T_{in,ac} } \right)}}{{A \times F \times \frac{{\left( {T_{st} - T_{in,ac} } \right) - \left( {T_{st} - T_{out,ac} } \right)}}{{\ln \left( {\frac{{T_{st} - T_{in,ac} }}{{T_{st} - T_{out,ac} }}} \right)}}}}$$

3 $$U_{clean} = {\frac{{\dot{v}_{ac,cir} \times \rho_{ac} \times Cp_{ac} \times \left( {T_{out,ac} - T_{in,ac} } \right)}}{{A \times F \times \frac{{\left( {T_{st} - T_{in,ac} } \right) - \left( {T_{st} - T_{out,ac} } \right)}}{{\ln \left( {\frac{{T_{st} - T_{in,ac} }}{{T_{st} - T_{out,ac} }}} \right)}}}}}$$

where $\acute{\upsilon }_{{{\text{ac}}}}$, ρ_ac_, Cp_ac_, T_in, ac_, T_out,ac_, T_st_, A and F are the volume flow rate, acid density, specific heat capacity of phosphoric acid, inlet and outlet temperatures of the phosphoric acid, steam temperature, heat transfer area and corrective factor for the average logarithmic temperature difference (= 1 pure Counter Flow Arrangement), respectively.

4 $$\dot{v}_{ac,cir} = f(HMT)$$

5 $$HMT = \frac{{P_{disch\arg e} - P_{suction} }}{{\rho_{ac} \times g}}$$

where HMT, P_discharge_ and P_suction_ are the total manometric head of the pump and discharge and suction pressures, respectively.

The second power transfer method is used to determining the uncertainty analysis on the measured data^15^. The relation between the dependent variable (Y) and independent variables (X_1_, X_2_, … X_n_) is given below :

6 $$Y = f(X_{1} ,X_{2} , \ldots ,X_{n} )$$

The uncertainty of variable Y is calculated by using the following equation:

7 $$dY = \sqrt {\sum\limits_{i = 1}^{n} {\left( {\frac{\delta Y}{{\delta X_{i} }}dX_{i} } \right)^{2} } }$$

where dX_i_ represents the uncertainties of each variable X_i_.

The fouling resistance (Rf), overall heat exchange coefficient (U) and total manometric head (HMT) are calculated using Eqs. (1–5). The relative uncertainty of such parameters are determined using the following equations^13^:

8 $$\frac{dRf}{{Rf}} = \sqrt {\left( {\frac{{dU_{fouling} }}{{U_{fouling} }}} \right)^{2} + \left( {\frac{{dU_{clean} }}{{U_{clean} }}} \right)^{2} }$$

9 $$\frac{dU}{U} = \sqrt {\left( {\frac{{d\dot{v}_{ac,cir} }}{{\dot{v}_{ac,cir} }}} \right)^{2} + \left( {\frac{{d\rho_{ac} }}{{\rho_{ac} }}} \right)^{2} + \left( {\frac{{dT_{in,ac} }}{{T_{in,ac} }}} \right)^{2} + \left( {\frac{{dT_{out,ac} }}{{T_{out,ac} }}} \right)^{2} + \left( {\frac{{dT_{st} }}{{T_{st} }}} \right)^{2} + \left( {\frac{{dT_{in,ac} }}{{T_{in,ac} }}} \right)^{2} + \left( {\frac{{dT_{st} }}{{T_{st} }}} \right)^{2} + \left( {\frac{{dT_{out,ac} }}{{T_{out,ac} }}} \right)^{2} }$$

10 $$\frac{dHMT}{{HMT}} = \sqrt {\left( {\frac{{dP_{suction} }}{{P_{suction} }}} \right)^{2} + \left( {\frac{{dP_{disch\arg e} }}{{P_{disch\arg e} }}} \right)^{2} + \left( {\frac{{d\rho_{ac} }}{{\rho_{ac} }}} \right)^{2} }$$

where ${\text{d}}\acute{\upsilon }_{{{\text{ac}},{\text{cir}}}}$, dρ_ac_, dT_st_, dT_in,ac_, dT_out,ac_, dP_suction_ and dP_discharge_ represent respectively the uncertainties related to the volume flow rate, density, steam temperature, acid inlet and outlet temperatures and suction and discharge pressures.

The uncertainties of collected parameters are listed in Table 1. The relative uncertainties of temperatures, density and pressure measurements are 0.3 °C, 0.05% rdg and 1.6% rdg, respectively. The relative uncertainties of Rf, U and HMT are within 8% in the entire experimental range.

### Principal component analysis (PCA) and step wise regression (SWR)

Principal Component Analysis (PCA) and Step Wise Regression (SWR) are one of the most powerful and more well-known approaches used to separate the variables influencing the dependent variable for modeling to reduce the data volume^17^. These two methods (PCA and SWR) were used in this study.

In the PCA approach, a linear combination of independent variables with the highest relationship with the dependent variable is determined, and usually this linear combination justifies a high percentage of changes in the dependent variable^18^.

In the SWR approach, the variables with the highest correlation with dependent variable are entered into the model. In the final step, a model containing a combination of the most influential variables is developed^17^.

The measurement ranges of input and output parameters used by PCA and SWR methods are presented in Table 2. The collected data consists of six operating parameters. The ranges of acid inlet temperature is between 68 and 78 °C, the acid outlet temperature is between 77 and 86.8 °C, the steam temperature is from 116 to 125 °C, acid density is between 1620 and 1656 kg/m^3^, acid volume flow rate is up to 3407 m^3^/h, and period time is between 0 and 122 h. The fouling resistance is from 0 to 0.00017 m^2^ °C /W. XLSTAT which is an additional component of Microsoft Excel was used to process the data matrix.

**Table 2:** Measurement ranges of parameters.

Input variables				Output variable			
Parameter		Min	Max	Parameter		Min	Max
t	Time	0	122 h	Rf	Fouling resistance	0	0.00017 m^2^°C/W
T_in,ac_	Acid inlet temperature	68 °C	78 °C				
T_out,ac_	Acid outlet temperature	77 °C	86.8 °C				
T_st_	Steam temperature	116 °C	125 °C				
ρ_ac_	Acid density	1620 kg/m^3^	1656 kg/m^3^				
$\acute{\upsilon }_{{{\text{ac}},{\text{cir}}}}$	Acid volume flow rate	2102 m^3^/h	3407 m^3^/h				

### Partial least squares (PLS) regression

Partial Least Squares (PLS) is a statistical regression method which is used to relate one response variable (Y) to a set of predictive variables (X_1_ … X_n_) by linear multivariate model^7,19^. The regression model was calculated according to the following equation:

11 $$Y = \alpha_{0} + \alpha_{1} X_{1} + \alpha_{2} X_{2} + \cdots + \alpha_{n} X_{n}$$

where Y is the response variable, α_0_ … α_n_ are the regression coefficients and X_1_. X_n_ are the input variables.

In our study, this technique is used to determine the relationship between operating variables collected from phosphoric acid concentration unit in order to predict the fouling resistance. The choice of using this technique is based on its predictive abilities and stability.

XLSTAT is used in this study to develop the PLS model. Time (t), acid inlet (T_in, ac_) and outlet (T_out, ac_) temperatures, steam temperature (T_st_), acid density (ρ_ac_) and acid volume flow $\left( {\dot{v}_{ac,cir} } \right)$ are the operating parameters used for modeling the fouling resistance (Rf). The ranges of these parameters is tabulated in Table 2.

### Artificial neural network (ANN) method

An Artificial Neural Network (ANN) approach was used in this study to estimate the fouling resistance in cross-flow heat exchanger by means of the operating data of the phosphoric acid concentration loop.

**Figure 2:**
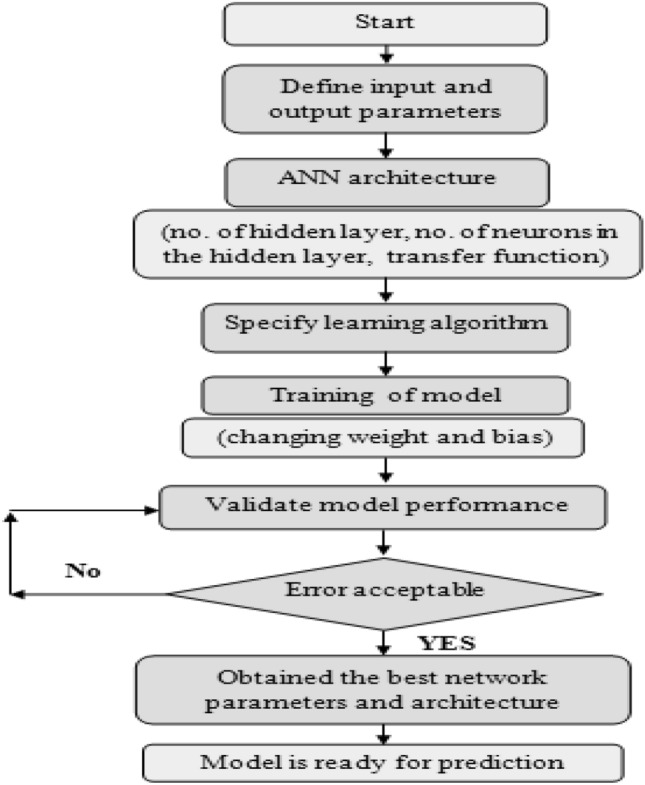
Flowchart of artificial neural network.

Figure 2 described the procedure used by ANN which is consists of three step which are^20–23^:

- *Collection of data and preprocessing.*
- *Building an artificial neural network* It includes the choice of training algorithm, activation function and the optimal number of neurons in hidden layer.
- *Train and evaluate the model obtained by using the full dataset* This step done after determining the best-performing structure of ANN.

Table 2 depicts the measurement ranges of input and output parameters used by ANN method. In the following section, feature variables and their impacts on the fouling resistance are investigated in more detail.

As mentioned previously, 361 experimental data was used to build the ANN model by using STATISTICA Software. The entire dataset in this study was randomly divided into three subsets: training set (70% of all data = 253 data), testing set (15% of all data = 54 data) and validation set (15% of all data = 54 data).

A Multi-Layer Perceptron (MLP) ANN model was built to predict the fouling resistance in cross-flow heat exchanger. In our case, back-propagation method is used to train the network. This method allowed to alter biases and weights in order to reduce the error between actual and predicted fouling resistance values.

**Figure 3:**
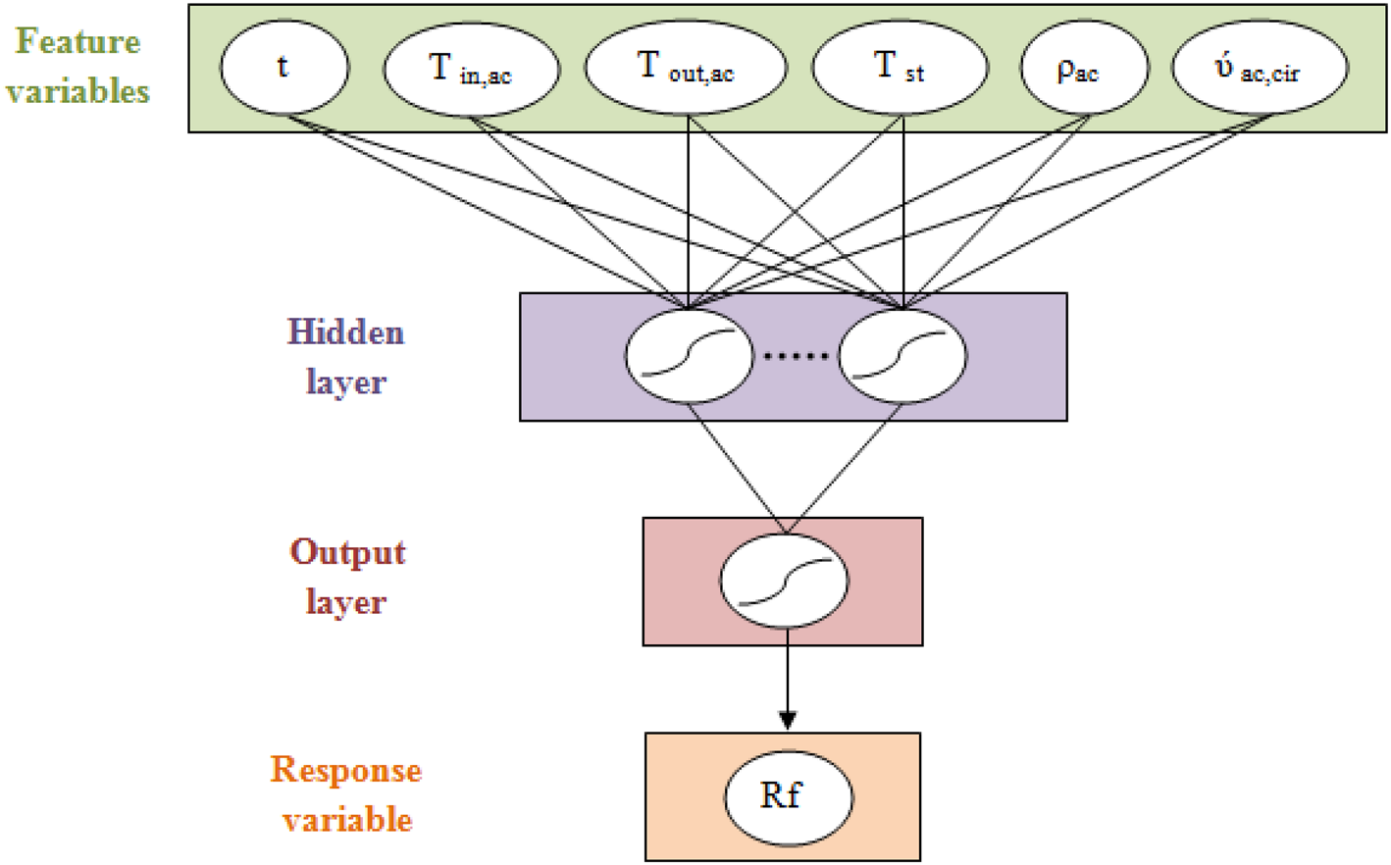
Architecture of MLP network model to predict fouling resistance.

The adequate ANN structure for fouling resistance prediction was determined by changing the number of neurons in the hidden layer (from 1 to 12 neurons), training algorithms (BFGS, gradient descent and conjugate gradient), transfer functions for hidden layer (tansig, purelin and sig), and the most effective network configuration was constructed (Fig. 3)^23,24^. The number of neurons, training algorithm and transfer function are determined based on the values of two statistical accuracy measurements which are the mean square error (MSE) and the correlation coefficient (r^2^)^23^.

Due to the STATISTICA software generates various random data for each run, the best ANN for each topology was chosen after a maximum of 30 runs.

After finding the best configuration of ANN method, a sensitivity analysis was investigated to reveal the usefulness of each operating variable, and also to identify the components that are most important for forecasting fouling resistance. For this, Eq. 12 was applied based on partitioning of connection weights anticipated by Garson^25,26^:

12 $$RI_{x} = \frac{{\sum\limits_{b = 1}^{{k_{h} }} {\left( {\left( {\frac{{\left| {W_{xb} } \right|}}{{\sum\limits_{a = 1}^{{k_{i} }} {\left| {W_{ab} } \right|} }}} \right) \times \left| {V_{b} } \right|} \right)} }}{{\sum\limits_{a = 1}^{{k_{p} }} {\left( {\sum\limits_{b = 1}^{{k_{h} }} {\left( {\left( {\frac{{\left| {W_{xb} } \right|}}{{\sum\limits_{a = 1}^{{k_{i} }} {\left| {W_{ab} } \right|} }}} \right) \times \left| {V_{b} } \right|} \right)} } \right)} }}$$

where RI is the relative importance of the input variable (x) on the output variable, k_i_ and k_h_ are the number of input and hidden neurons respectively, W_ab_ are the connection weights between the input layer and the hidden layer, V_b_ is the connection weight between the hidden layer and the output layer.

It should be noted that the numerator in the Eq. (12) describes the sum of the products of the absolute weights for each input. However, the denominator represents the total of all the weights feeding the hidden unit, taking the absolute values.

### Efficacy of models

Two statistical quality parameters which are mean squared error (MSE) and correlation coefficient (r^2^) were used in this study to objectively examine the efficiency of PLS and ANN models to predict the fouling resistance in cross flow heat exchanger. The following equations gives the mathematical expressions of MSE and r^2^^6,9^:

13 $$MSE = \frac{1}{M}\sum\limits_{j = 1}^{M} {(Rf_{j} - Rf_{j}^{pred} )^{2} }$$

14 $$r^{2} = \frac{{\sum\limits_{j = 1}^{M} {\left( {Rf_{i} - \left\langle {Rf} \right\rangle } \right)^{2} - \sum\limits_{j = 1}^{M} {(Rf_{j} - Rf_{j}^{pred} )^{2} } } }}{{\sum\limits_{j = 1}^{M} {\left( {Rf_{j} - \left\langle {Rf} \right\rangle } \right)^{2} } }}$$

where M is the number of data, Rf, Rf ^pred^ and $\left\langle {Rf} \right\rangle$ denote the observed values, the anticipated values and the average values of the fouling resistance, respectively.

## Results and discussions

### Principal component analysis (PCA) and stepwise regression (SWR)

As mentioned previously, two powerful methods which are Principal Component Analysis (PCA) and Step Wise Regression (SWR) were used with the aim of creating an adequate model to predict fouling resistance based on operating variables collected from phosphoric acid concentration unit.

**Figure 4:**
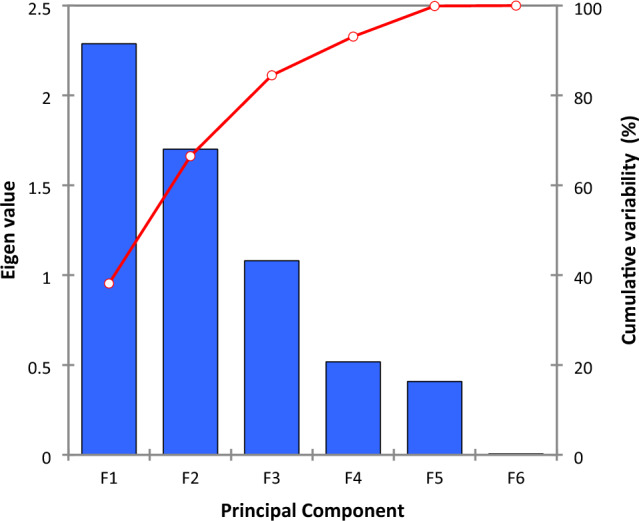
Eigen and cumulative variability of principal components.

**Figure 5:**
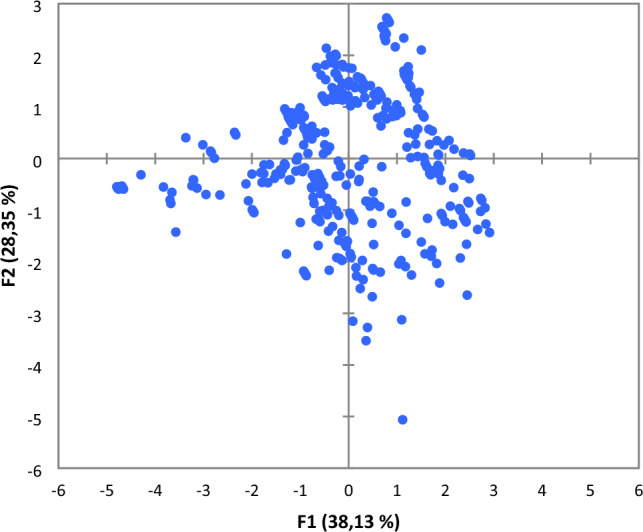
Score plot of the first and second principal components F1 and F2.

**Figure 6:**
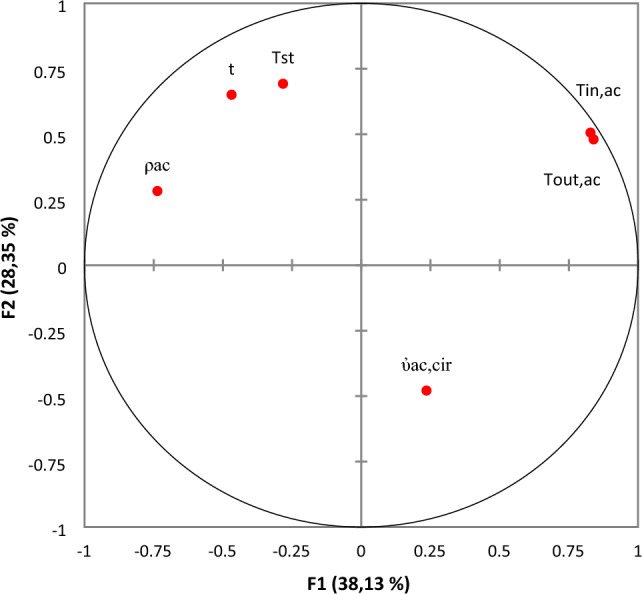
Loading plot of the two principal components F1 and F2.

PCA results for eigen-values and cumulative variables, score plot and corresponding loading plot are displayed in Figs. 4, 5 and 6, respectively.

As can be seen from Fig. 4, the first three components (F1 (time), F2 (acid inlet temperature) and F3 (acid outlet temperature)) account 38.13%, 28.35% and 18% respectively of the original matrix information. The two components (F1 and F2) explain 66.47% and the three components contributed for more than 84% of the variation. The remaining components (F4 (steam temperature), F5 (acid density) and F6 (volume flow rate)) account all 15.5%.

PCA of score plot of the two first principal component shown in Fig. 5 confirms the normal functioning of the phosphoric acid concentration unit during the studied period due to the clusters of observations are located in the center of the score plot.

PCA of corresponding loading plot of the first two components (F1 and F2) is displayed in Fig. 6. As can be seen from this figure, the inlet and outlet temperatures of phosphoric acid have a positive influence on both Fs. Volume flow rate has a positive and a slight negative influence, following F1 and F2, respectively. It should be noted that there is no variable that don’t contribute to the whole process (has zero weight).

Based on SWR, the six attributes (t, T_in,ac_, T_out,ac_, T_st_, ρ_ac_ and $\acute{\upsilon }_{{{\text{ac}},{\text{cir}}}}$) were incorporated in the model (Table 3). The results presented in this table confirmed that time and acid inlet and outlet temperatures are the most contributed parameters of the variation.

**Table 3:** Stepwise regression analysis for fouling resistance as the dependent variable.

Step	Entered variable	Variables in model	Model R-square	Model adjusted R-square
1	$\acute{\upsilon }_{{{\text{ac}},{\text{cir}}}}$	$\acute{\upsilon }_{{{\text{ac}},{\text{cir}}}}$	0.047	0.044
2	ρ_ac_	$\acute{\upsilon }_{{{\text{ac}},{\text{cir}}}}$, ρ_ac_	0.136	0.131
3	T_st_	$\acute{\upsilon }_{{{\text{ac}},{\text{cir}}}}$, ρ_ac_, T_st_	0.398	0.393
4	T_out,ac_	$\acute{\upsilon }_{{{\text{ac}},{\text{cir}}}}$, ρ_ac_, T_st_, T_out,ac_	0.461	0.454
5	T_in,ac_	$\acute{\upsilon }_{{{\text{ac}},{\text{cir}}}}$, ρ_ac_, T_st_, T_out,ac_, T_in,ac_	0.975	0.975
6	t	$\acute{\upsilon }_{{{\text{ac}},{\text{cir}}}}$, ρ_ac_, T_st_, T_out,ac_, T_in,ac_, t	0.985	0.985

Based on the results achieved from both PCA and SWR techniques, the attributes (t, T_in,ac_, T_out,ac_, T_st_, ρ_ac_ and $\acute{\upsilon }_{{{\text{ac}},{\text{cir}}}}$) were selected to be the most proper input parameters for both the PLS and ANN models.

### Partial least squares (PLS) regression

In our PLS model, the X matrix is composed by the 6 variables collected during the phosphoric acid concentration process, which are listed in Table 2. However, the Y response is the fouling resistance of the heat exchanger (Rf).

**Figure 7:**
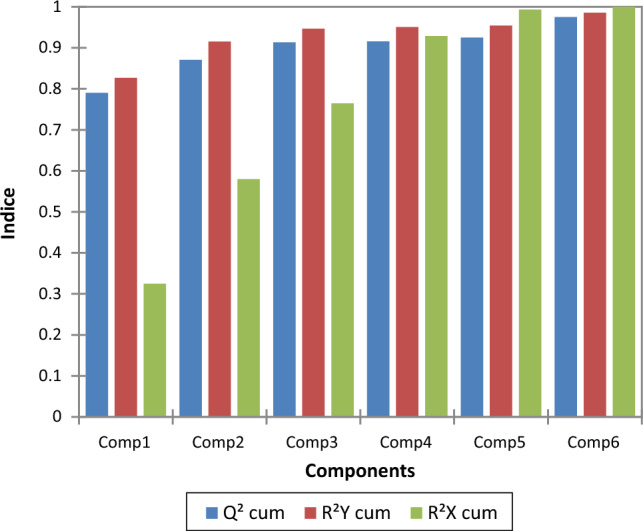
Quality of PLS model by number of components. Loading plot of the two principal components F1 and F2.

The quality of PLS model for the six components is displayed in Fig. 7. As can be seen from this figure, the values of Q^2^_cum_, R^2^Y_cum_ and R^2^X_cum_ for the two principal components (F1 and F2) are equal to 0.871, 0.915 and 0.578, respectively. These results confirms that the optimal balance between fit and predictive ability of the computed model is guaranteed by the two first components.

**Figure 8:**
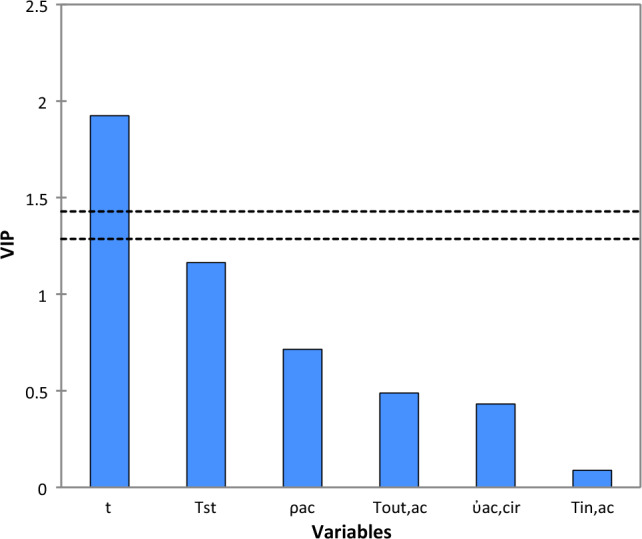
Variable importance in the projection for PLS model for 2 principal components and 95% confidence interval.

The contribution of each input variable in the prediction of fouling resistance in a descending order is depicted in Fig. 8. As can be seen from this figure, the variables time (t) and steam temperature (Tst) have the highest impact on the fouling resistance (Rf). The values of variable importance in the projection (VIP) for the two input variables are respectively equal to 1.9241 and 1.1640.

The statistical parameters values (MSE and r^2^) are shown in Table 4 for the PLS model. The high (r^2^) value (0.992) near to unity and the lowest value of (MSE) indicated satisfactory adjustment of the PLS model to the experimental results.

**Table 4:** Summary of statistical parameters values for PLS model.

Parameter	PLS
MSE	2.607 × 10^–11^
r^2^	0.992

**Figure 9:**
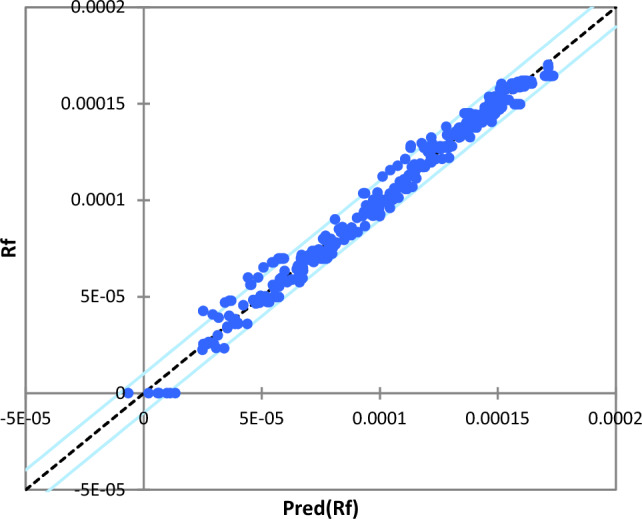
Comparison between actual and predicted heat exchanger fouling resistance by PLS model.

A comparison between the actual fouling resistance and the predicted fouling resistance is displayed in Fig. 9. As can be seen from this figure, the concentration of the set observations in the line y = x affirms the good agreement of the PLS model with the experimental data.

### Artificial neural network (ANN)

#### Correlation matrix analysis

It will begin with determining the strength of the relation between the response and feature variables. For this purpose, the degrees of relevancy between fouling resistance and the considered feature variables are calculated by Pearson’s correlation and presented in Table 5^27^. The results reveals that time, steam temperature and acid density indirectly affect the response variable. Moreover, time and steam temperature have the most direct influence, and acid outlet temperature has the most indirect effect on the fouling resistance.

**Table 5:** Degrees of relevancy between the fouling resistance and feature variables.

Feature variables	Person’s coefficient
Time	0.964
Acid inlet temperature	− 0.044
Acid outlet temperature	− 0.244
Steam temperature	0.583
Acid density	0.358
Acid volume flow rate	− 0.216

#### Finding the best configuration

Table 6 demonstrates that the training algorithm and transfer function contributed significantly to the total variance in ANN efficiency.

**Table 6:** Comparison of errors of various algorithm and transfer function for estimation of Rf.

Algorithm	Transfer function	MSE _training_	MSE _validation_	MSE _test_	r^2^_training_	r^2^_validation_	r^2^_test_
BFGS	Hyperbolic tangent sigmoid	1.668 × 10^−11^	2.585 × 10^−11^	1.707 × 10^−11^	0.995	0.993	0.995
	Linear	4.221 × 10^−10^	3.374 × 10^−10^	4.595 × 10^−10^	0.864	0.911	0.865
	Sigmoid	4.246 × 10^−10^	4.646 × 10^−10^	4.513 × 10^−10^	0.866	0.874	0.866
Gradient descent	Hyperbolic tangent sigmoid	6.833 × 10^−11^	5.966 × 10^−11^	5.209 × 10^−11^	0.982	0.986	0.986
	Linear	1.036 × 10^−09^	7.187 × 10^−10^	1.186 × 10^−09^	0.786	0.911	0.735
	Sigmoid	4.400 × 10^−10^	4.563 × 10^−10^	4.798 × 10^−10^	0.863	0.879	0.862
Conjugate gradient	Hyperbolic tangent sigmoid	4.052 × 10^−11^	5.628 × 10^−11^	4.422 × 10^−11^	0.988	0.986	0.990
	Linear	4.478 × 10^−10^	3.271 × 10^−10^	4.681 × 10^−10^	0.859	0.914	0.862
	Sigmoid	4.349 × 10^−10^	4.625 × 10^−10^	4.545 × 10^−10^	0.864	0.876	0.865

According to the obtaining results, it should be noticed that the BFGS back-propagation and the hyperbolic tangent sigmoid transfer function are respectively the most appropriate training algorithm and activation function. For validation data, the MLP developed model have the smallest MSE value (2.585 × 10^–11^) and the highest r^2^ value (0.993). Table 7 illustrates the performance results of developed neural network for training data, testing data, validation data and all data. The optimal ANN structure is composed by 6 neurons in the hidden layer and the hidden and output layers have a tangent sigmoid transfer function.

**Table 7:** Performance of developed neural network.

Neural network structure	r^2^_training_	r^2^_validation_	r^2^_test_	r^2^_all_	MSE _training_	MSE _validation_	MSE _test_	MSE _all_	Algorithm	Hidden activation function	Output activation function
6-6-1	0.995	0.993	0.995	0.995	1.668 × 10^−11^	2.585 × 10^−11^	1.707 × 10^−11^	1.811 × 10^−11^	BFGS	Tanh	Tanh

**Figure 10:**
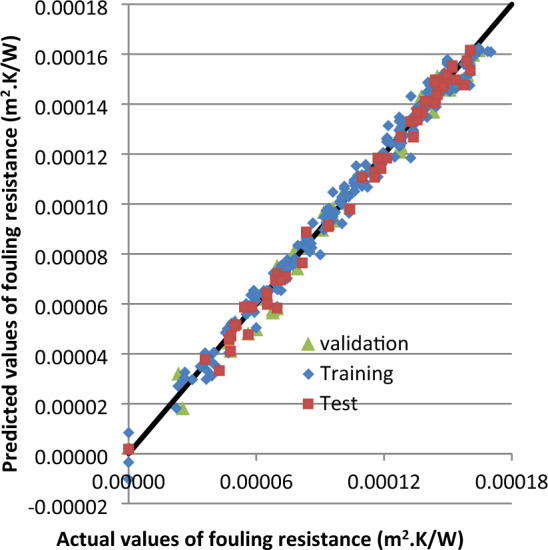
Comparison of actual and predicted Rf on the validation, training and test dataset.

The comparison between the experimental datasets of the fouling resistance and the corresponding estimated values of the network for training, testing, and validation data set in Fig. 10 indicates the high rate of precision of ANN method.

**Figure 11:**
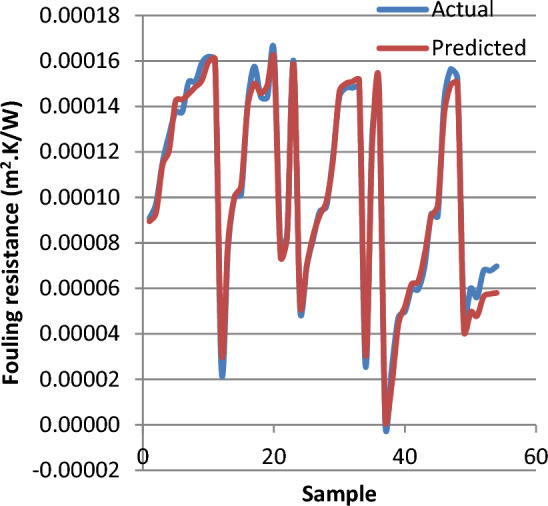
Comparison of actual and predicted Rf on the validation dataset.

**Figure 12:**
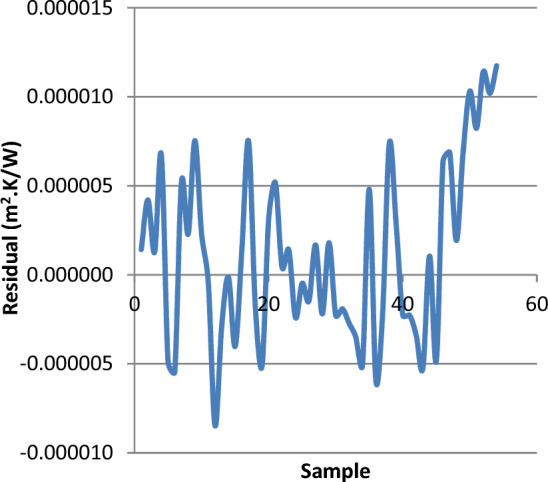
Residual Rf, estimation on the validation dataset.

For better visualization, Figs. 11 and 12 show a comparison between the experimental data sets and ANN predicted data of fouling resistance in heat exchanger and the residual on the validation dataset. These figures confirm an excellent prediction performance of ANN method.

#### Sensitivity analysis of artificial neural network (ANN)

Table 8 gives the obtained values of the weights and the biases (W_ab_, V_b_, b_b_, and b_out_) for the optimal ANN structure given in Table 7. The values of neural network weights are used to know the relative importance of the different input variables (time, acid inlet and outlet temperatures, steam temperature, density and volume flow rate) on the output variable (fouling resistance).

**Table 8:** Optimal values of weights and biases obtained during training of ANN.

	b	1	2	3	4	5	6
Weights between the input and the hidden layers	W_1b_	1.602	− 1.38	− 1.139	− 0.35	− 2.104	1.252
	W_2b_	0.172	− 0.342	− 1.362	0.68	0.464	0.2
	W_3b_	− 0.393	− 0.181	1.873	− 0.644	0.56	− 0.374
	W_4b_	0.023	− 0.73	− 0.426	0.082	− 0.527	0.761
	W_5b_	0.359	− 0.447	0.118	0.082	− 0.113	0.263
	W_6b_	− 0.055	0.023	0.252	− 0.475	− 0.587	0.234
Bias values of hidden layer	b_b_	− 0.501	− 0.352	0.919	0.551	− 0.758	0.026
Weights between the hidden and the output layers	V_b_	− 0.397	− 0.396	− 2.198	2.51	− 0.956	− 0.6
Bias values of output layer	b_out_	0.405					

**Figure 13:**
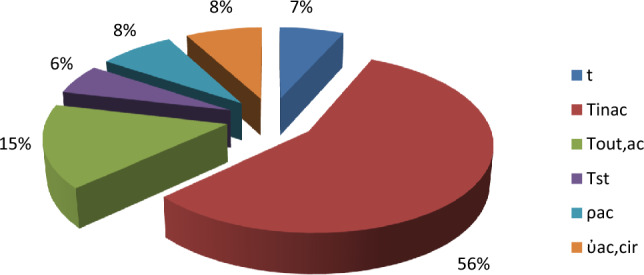
Relative importance of input variables on output.

The relative importance of input variables on fouling resistance is determined by using the results obtained in Table 8 and by applying Garson equation (Eq. 12). Figure 13 illustrates a summary of the obtained results. It can be seen that the acid inlet and outlet temperatures have the highest impacts on the fouling resistance value with importance equal to 56% and 15.4%, respectively, but density, volume flow rate, time and steam temperature have approximately same impact on the fouling value with an importance equal to 7.7%, 7.8%, 6.6% and 5.6%, respectively.

### Models performance

In this work, 361 experimental data were used to modeling the fouling resistance by using two different techniques which are partial least squares regression and artificial neural network. As can be seen from Table 7, the optimum structure of the ANN model reaches MSE = 2.585 × 10^−11^ and r^2^ = 0.993. For the PLS model, the values of MSE and r^2^ are equal to 2.607 × 10^−11^ and 0.992, respectively, as shown in Table 4. These statistical parameters indicated that the values of r^2^ and MSE predicted by ANN are comparatively closer respectively to 1 and 0 than PLS method which implies that model developed by ANN estimated the fouling resistance more precisely than PLS method.

The developed ANN and PLS models are specific to a type of fluid which is the phosphoric acid. This fluid contained impurity and suspended solids. The two models are applicable to a system of variables within the permitted ranges as shown in Table 2. For operating periods ranging up to 122 h, the inlet and outlet temperatures of fluid and the steam temperature should not exceed 78 °C, 86.8 °C and 125 °C, respectively, the phosphoric acid density should not be above 1656 kg/m^3^, and the volume flow rate should not be below 2102 m^3^/h to obtain values of (Rf) close to reality by the developed models.

A second comparison between the accuracy measurements of the developed ANN model in this work and other studies which used ANN method to modeling the fouling resistance in the petroleum and chemical industries and in water treatment are shown in Table 9^28^.

**Table 9:** Studies on ANN fouling modeling.

Author	Accuracy measurements
Davoudi and Vaferi^29^	AARD = 5.42% MSE = 0.0013 RMSE = 0.0355 r^2^ = 0.977819
Biyanto^30^	RMSE = 1.38 × 10^−5^
Benyekhlef^31^	MAP E = 2.3714% MSE = 6.2119e−004 RMSE = 0.0249 R^2^ = 0.9
Peleato et al.^32^	Mean absolute relative error < 5%
Soleimani et al.^33^	R^2^ = 0.9999
Salehi et al.^34^	r^2^ = 0.98
Shetty et al.^35^	Absolute relative error < 5%

The specific characterization of this work is the fouling element used (phosphoric acid) as well as the main function of heat exchanger in phosphoric acid concentration plant.

According to Table 9, it can be concluded that the obtained ANN model is significantly better than other ANN fouling models. The accuracy measurements of the obtained model are less than 0 for MSE and closest to 1 for r^2^ in comparison with the values of other models.

## Conclusion

In the current study, the fouling resistance in cross flow heat exchanger was modeled using linear and non linear method based on the operating variables collected from phosphoric acid concentration unit. The data set was processed using PCA and SWR in order to determine the highest impacts of process parameters on the fouling resistance. Then, a PLS model was developed based on the input matrix to predict the fouling resistance. The precision measurements of the linear model obtained by PLS method with the current results reflect a good agreement. To enhance the accuracy performance of the linear model, an ANN model was used to estimate fouling resistance.

Several networks with different algorithm and transfer function were compared and assessed based on two statistical measurements. The optimal training data was attained with 6-6-1 structure considering the BFGS back-propagation training algorithm and the tangent sigmoid transfer function in the hidden and output layers. Mean squared error (MSE) of 1.811 × 10^−11^ and correlation coefficient (r^2^) of 0.995 were obtained by the ANN model for all data sets.

Based on ANN results, sensitivity analysis was determined. It was noticed that the acid inlet and outlet temperatures have the highest impacts on the fouling resistance. The implementation of the developed models onsite could achieve the stability of the operation plant and significant savings.

## Data Availability

The datasets used and/or analyzed during the current study available from the corresponding author on reasonable request.
